# UV Irradiation as a Versatile Low‐Temperature Strategy for Fabricating Templated Mesoporous Titania Films

**DOI:** 10.1002/smll.202409856

**Published:** 2024-12-17

**Authors:** Guangjiu Pan, Shanshan Yin, Linus F. Huber, Zerui Li, Ting Tian, Lukas V. Spanier, Huaying Zhong, Tianfu Guan, Caroline R. Ehgartner, Nicola Hüsing, Matthias Schwartzkopf, Stephan V. Roth, Peter Müller‐Buschbaum

**Affiliations:** ^1^ Chair for Functional Materials Department of Physics TUM School of Natural Sciences Technical University of Munich James‐Franck‐Str. 1 85748 Garching Germany; ^2^ School of Mathematics and Physics Jiangsu University of Technology Changzhou 213001 China; ^3^ Chemistry and Physics of Materials Paris‐Lodron University Salzburg Jakob‐Haringer Straße 2a Salzburg 5020 Austria; ^4^ Deutsches Elektronen‐Synchrotron DESY Notkestraße 85 22607 Hamburg Germany; ^5^ Division of Coating Technology KTH Royal Institute of Technology Teknikringen 48 Stockholm 100 44 Sweden

**Keywords:** block copolymers, low‐temperature synthesis, mesoporous, thin films, titania

## Abstract

Mesoporous titania thin films offer promising applications in sensors, batteries, and solar cells. The traditional soft templating methods rely on high‐temperature calcination, which is energy‐intensive, incompatible with thermosensitive flexible substrates, and destructive for titania structures. This work demonstrates UV irradiation as a versatile low‐temperature and energy‐saving alternative for mesoporous crystalline titania fabrication. Grazing incidence wide‐angle X‐ray scattering analysis reveals a three‐stage crystallization process with increasing UV irradiation time supported by photoluminescence data. UV‐irradiation‐derived samples exhibit crystallinity and crystal size comparable to that of calcination. Integration with block copolymer templated sol–gel synthesis enables the creation of various morphologies, including cylindrical, ordered spherical, and hybrid structures. Characterizations via scanning electron microscopy and grazing incidence small‐angle X‐ray scattering confirm the homogeneity of morphology in the resulting films. The resulting films maintain similar optical properties despite morphological differences, as demonstrated by photoluminescence and UV–vis measurements. The versatility of UV irradiation extends to different titanium precursors, underscoring it as a flexible and efficient method for mesoporous titania thin film fabrication at low temperatures.

## Graphical abstract

UV irradiation is introduced as a versatile, low‐temperature alternative to traditional calcination for synthesizing high‐quality mesoporous crystalline titania. This method achieves crystallinity and crystal sizes comparable to calcination without causing structure collapse. It is compatible with block copolymer templated sol–gel synthesis and applicable to various titanium precursors, enabling efficient thin‐film production with diverse morphologies.

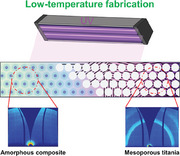

## 1 Introduction

Mesoporous materials show structure‐dependent advantages by means of their high surface area, tunable pore size, shape and distribution, and diverse compositions. These properties endow mesoporous materials with applications in catalysis, sensors, absorption and separation, batteries, solar cells, and drug delivery.^[^ ^1^, ^2^, ^3^ ^]^ Various mesoporous materials, including carbon, metals, metal oxides, ceramics, etc., have been synthesized. Among these materials, mesoporous metal oxides, especially mesoporous titania, are of great interest due to their functional properties and industrial application potential. Mesoporous titania has been applied in solar cells, photocatalysis and photoelectrochemistry, batteries, coatings, and molecule separations.^[^ ^4^, ^5^ ^]^ The typical synthesis strategies of mesoporous titania include template‐free, soft‐templating, and hard‐templating approaches. Due to the high flexibility, general applicability, and much better control over porous structures, the soft templating method has been widely used to fabricate well‐defined mesoporous titania.^[^ ^6^, ^7^, ^8^ ^]^ The obtained films from this soft templating method are composites of soft templates and amorphous titania and require post‐treatments, such as generally calcination at 300–500 °C, to remove the soft templates and crystallize the amorphous titania. This high‐temperature calcination process is energy‐consuming, incompatible with thermosensitive flexible substrates, and destructive for titania structures of small scale.^[^ ^9^, ^10^ ^]^ A low‐temperature fabrication process is intriguing for energy‐saving and applications on flexible substrates.

Various strategies have been developed to fabricate crystalline mesoporous titania at low temperatures. Hydrothermal treatment,^[^ ^11^, ^12^, ^13^ ^]^ metal‐induced crystallization,^[^ ^14^, ^15^, ^16^ ^]^ and photochemical processes^[^ ^17^ ^]^ have been used to synthesize crystalline titania at low temperatures. However, these methods often encounter challenges such as incomplete polymer template removal, structural changes or collapse,^[^ ^11^, ^12^, ^13^ ^]^ inhomogeneous film crystallization,^[^ ^14^ ^]^ or reliance on specific precursor designs.^[^ ^17^ ^]^ Other techniques, such as laser sintering, microwave treatment, and ultrasound irradiation, are proposed to fabricate crystalline titania via different energy inputs.^[^ ^18^, ^19^, ^20^ ^]^ They are efficient for nanoparticles and compact films but still challenging for applications on mesoporous titania thin films due to the delicate balance between template removal, crystalline materials, and structure maintenance. UV light irradiation has been proven to be an effective post‐synthetic method, potentially replacing high‐temperature calcination.^[^ ^21^, ^22^ ^]^ The UV light with a shorter wavelength interacts with oxygen in the ambient environment and generates reactive oxygen species to remove organic species in the films. Besides, with strong absorption in the UV region, the titania is transformed into a crystalline phase via UV irradiation.^[^ ^23^ ^]^ By utilizing UV irradiation, polymer removal, and titania crystallization are achieved. However, previous studies mainly focused on applications of UV irradiation on nanoparticle synthesis or employing special precursors.^[^ ^24^, ^25^, ^26^, ^27^ ^]^ These applications rely on special precursors or aqueous environments to induce low‐temperature crystallization. This limitation constrains the potential application of UV irradiation‐assisted low‐temperature fabrication.

In the present study, we demonstrate that UV irradiation is a versatile and powerful post‐synthetic method to fabricate crystalline mesoporous titania thin films. Integrating with the soft templating approach, various morphologies are accessible with crystalline phases from different titanium precursors. In this work, mesoporous titania films are prepared by block copolymer (BCP) templated sol–gel synthesis. The BCP poly(styrene‐block‐ethylene oxide) (PS‐*b*‐PEO) is the soft template. Ethylene glycol‐modified titanate (EGMT), a solid crystalline precursor, is used as a model system for sol–gel synthesis. Grazing incidence wide‐angle X‐ray scattering (GIWAXS) measurements reveal the crystal structure of the titania films, and the mesostructures after UV irradiation are probed via scanning electron microscopy (SEM) and grazing incidence small angle X‐ray scattering (GISAXS). The optoelectronic properties of the mesoporous titania are examined by photoluminescence (PL) spectroscopy and UV–vis spectroscopy. The general applicability of UV irradiation‐assisted low‐temperature fabrication is expanded to other commercial titanium precursors beyond EGMT.

## 2 Results and Discussion

### 2.1 Fabrication of Mesoporous Titania at Low Temperature

**Figure 1:**
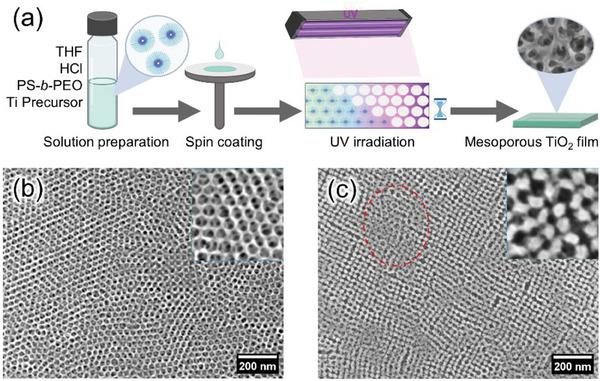
(a) Sketch of UV irradiation‐mediated low‐temperature fabrication of mesoporous titania; this process includes the preparation of sol–gel solution, film deposition via spin coating, and UV irradiation. SEM images of samples after (b) UV irradiation and (c) calcination at 400 ^○^C; the top‐right insets refer to the zoom‐in photos of local areas. The red dashed circle highlights the structure collapse of titania mesostructures caused by calcination.

BCP templated sol–gel synthesis is used to prepare mesoporous titania films (**Figure** **1**a). PS‐*b*‐PEO is the soft template with PS as the core and PEO as the corona in the mixture of tetrahydrofuran/hydrochloric acid (THF/HCl).^[^ ^28^ ^]^ Titania nanoparticles are generated via the hydrolysis and condensation of titanium precursors. These hydrophilic titania nanoparticles selectively interact with the PEO block and assemble with BCP during deposition. The obtained polymer/titania composites are treated with either UV irradiation or calcination to remove the polymer and produce a crystalline phase. UV irradiation in an ambient environment leads to photolysis degradation of polymer templates (Figures S1–S3, Supporting Information). The photon energy of the UV light is above 298 kJ.mol^−1^, which is sufficient to break bonds in the polymer backbone, such as C─C and C─O, which have bond energies of 348 and 360 kJ.mol^−1^, respectively, thereby initiating photodegradation.^[^ ^29^ ^]^ In addition, the high‐energy photons interact with oxygen and water in the air, producing oxygen and hydroxyl radicals.^[^ ^30^, ^31^ ^]^ These radicals and the UV irradiation decompose polymer chains. Previous studies showed that the photolysis of PEO predominantly involves the cleavage of ether bonds (C─O─C), forming radicals that subsequently lead to ester structures or random degradation products.^[^ ^32^ ^]^ Similarly, the degradation of PS under UV in the air follows a stepwise radical mechanism: (C‐H) → (C‐O) → (C = O) → (C‐OH) → (O‐C = O).^[^ ^33^ ^]^ The FTIR data show good consistency with these previous studies (Figure S2b, Supporting Information). Figure 1b,c show the SEM images of mesoporous titania after UV irradiation and calcination, respectively. The polymer template is removed by UV irradiation or calcination. However, high‐temperature calcination results in structure collapse and severe aggregation of the titania framework (Figure 1c), a common problem in the calcination process.^[^ ^34^ ^]^ In contrast, the frame of mesoporous titania is well preserved after UV irradiation (Figure 1b). This low‐temperature method has the potential for industrial applications and is compatible with thermosensitive flexible substrates, as demonstrated with slot‐die‐coated titania films on indium tin oxide‐coated polyethylene terephthalate (ITO‐coated PET, Figure S4, Supporting Information). The comparison of the energy consumption between UV irradiation and calcination reveals that UV irradiation is significantly more energy‐efficient (Table S1, Supporting Information). Accordingly, UV irradiation is used to replace high‐temperature calcination as a low‐temperature and energy‐saving alternative in the present study. The crystallization kinetics of titania under UV irradiation, accessible morphologies, and optoelectronic properties are investigated with EGMT as a model system. The general applicability of this low‐temperature process is then expanded to other commercial titanium precursors.

### 2.2 Crystallization Kinetics

**Figure 2:**
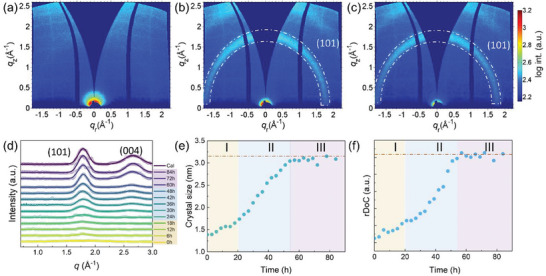
UV irradiation mediated low‐temperature crystallization revealed by GIWAXS. 2D GIWAXS data of (a) pristine sample, (b) UV irradiated sample, and (c) calcined sample. The white dashed rings highlight the (101) signal of anatase. d) Selected *pseudo*‐XRD data extracted from 2D GIWAXS data for different UV irradiation times; the raw data are plotted with hollow circles, and the Gaussian fit results are plotted as solid lines. All data and fits are shifted vertically for presentation. e) Crystal size evolution and (f) *rDoC* over irradiation time; the values of crystal size and *rDoC* from the calcined sample are indicated as orange dashed lines in (e) and (f). The crystallization process is divided into three stages: stage I (0–20 h), stage II (20–54 h), and stage III (54 h‐), as indicated by light yellow, blue, and purple, respectively.

The titania prepared from the sol–gel method is generally amorphous. In the sol–gel synthesis, the titania alkoxides undergo hydrolysis to release the alcohol and form hydrophilic hydroxy titanium monomers. These released monomers bridge with each other and grow to oligomers or even nanoparticles, which is polycondensation and can happen at the same time as hydrolysis. The resulting materials are amorphous in nature (**Figure** **2**a). Previous studies showed that the amorphous titania has an electronic and local molecular structure similar to anatase.^[^ ^35^ ^]^ Both five‐ and six‐coordinated titanium atoms were present in amorphous titania with a core‐shell structure, in which the surface shells were TiO_5_ units and the anatase‐like crystalline cores were TiO_6_ units.^[^ ^35^, ^36^, ^37^ ^]^ For mesoporous amorphous titania, the five‐ and six‐coordinated titanium atoms account for 39% and 61%, respectively.^[^ ^38^, ^39^ ^]^ The building blocks TiO2‐ 6 octahedra of the amorphous titania are randomly linked together and lack long‐range ordering, as indicated by the absence of pronounced scattering features in the 2D GIWAXS data (Figure 2a). During UV irradiation, the inorganic components absorb the high‐energy photons of UV light, triggering electronic excitation and activation that facilitates dehydration, densification, and crystallization.^[^ ^17^, ^31^, ^40^, ^41^, ^42^ ^]^ After UV irradiation, two bright rings appear in the 2D GIWAXS data (Figure 2b), which are very similar to the data from the calcined sample (Figure 2c). The *d*‐spacings of UV irradiated and calcined samples calculated from these two rings are 3.51 and 2.36 Å, coming from (101) and (004) signals from anatase, respectively. Accordingly, we find anatase with lattice constants *a* = *b* = 3.78 Å and *c* = 9.44 Å with a tetragonal structure (Figure S5, Supporting Information). The formation of anatase is anticipated since the anatase phase is more thermodynamically stable than rutile when the crystallite size of nanocrystals is smaller than 11 nm.^[^ ^43^ ^]^ A slight orientation is present in these anatase crystals with a stronger intensity near *χ =* 0° in the GIWAXS data, which may be associated with the interface‐mediated alignment of the crystals in the thin film geometry. The similarity of the 2D GIWAXS data indicates the effectiveness of UV irradiation as a low‐temperature method for deriving crystalline titania compared to the classical calcination process.

The *selected pseudo*‐XRD data (Figure 2d) extracted from 2D GIWAXS data show the crystallization kinetics over different UV irradiation times. The evolutions of crystal size (Figure 2e) and relative degree of crystallinity (*rDoC*, Figure 2f) are extracted from *pseudo*‐XRD data. According to Figure 2d–f, the crystallization process can be divided into three stages, namely stage I (0–20 h), stage II (20–54 h), and stage III (54 h‐). In stage I, no prominent diffraction peak is observed in the *pseudo*‐XRD patterns. The analysis of crystal size and *rDoC* indicates the presence of small nano‐crystallites in the amorphous sample obtained from sol–gel synthesis. In the first stage, the crystal size and *rDoC* increase slowly. These nano‐crystallites serve as nuclei during crystallization in the later stages. After forming many nuclei in stage I, the crystal size and *rDoC* increase dramatically in stage II. At stage II, a prominent peak at *q* = 1.79 Å^−1^ arises first, followed by another peak at *q* = 2.66 Å^−1^. These two peaks evolve narrower and stronger with increasing UV irradiation time, indicating larger crystallite sizes and more crystallites. During stage III, the films are stable with a constant crystal size and *rDoC*. After 54 h of UV irradiation, the crystal size stops growing. This behavior might result from the structure constraint of mesostructures. The calcined sample has a relatively larger crystal size, as indicated by the orange dashed line in Figure 2e. The crystal size and *rDoC* of samples with UV irradiation are comparable with those from the calcined sample, indicating UV irradiation as a promising low‐temperature calcination alternative.

**Figure 3:**
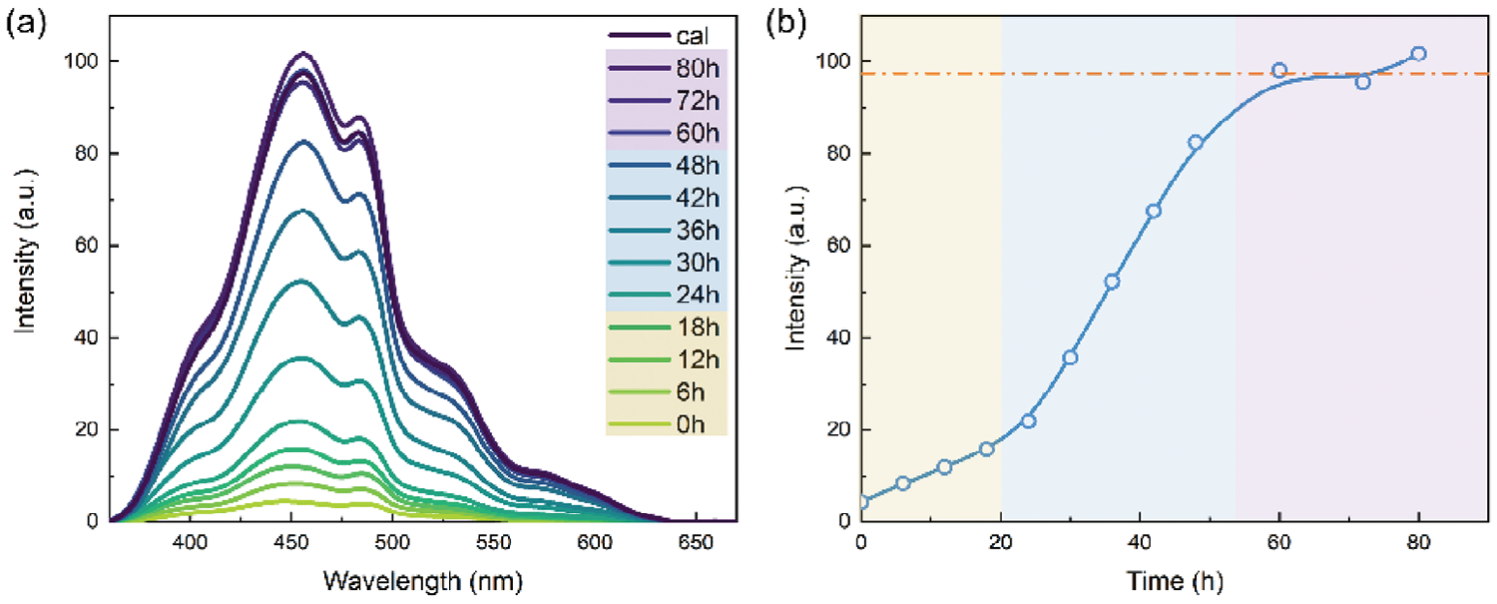
PL evolution during UV irradiation mediated low‐temperature crystallization. a) PL emission spectra evolution of mesoporous titania under different UV irradiation times; the excitation wavelength is 320 nm. b) Temporal PL intensity evolution at 456 nm. The crystallization process is divided into three stages: stage I (0–20 h), stage II (20–54 h), and stage III (54 h‐), as shadowed by light yellow, blue, and purple, respectively. The value from the calcined sample is presented as the orange dashed line.

The evolution of the PL spectra can also verify the crystallization process. **Figure** **3**a,b show the PL spectra and peak intensity evolution of mesoporous titania with different irradiation times. The deconvolution of the PL spectra is carried out by Gaussian fits (Figure S6, Supporting Information), and the attributions of different defects are summarized in Table S2 (Supporting Information). The PL intensity at 456 nm is used to track the PL intensity evolution. The evolution of the PL spectra shows a similar trend to that of GIWAXS data. The pristine film is amorphous with nanocrystallites, yielding very weak PL intensity. The PL intensity increases slowly in the first stage, then rapidly in the second stage, and stays stable at the last stage. It is worth mentioning that the peaks of PL spectra remain the same for all time due to the fact that the signal mainly comes from the crystalline phase of titania, and the amorphous materials contribute little to the PL intensity.

UV‐irradiation‐induced crystallization is associated with photoexcitation, driving the rearrangement of these octahedral units. The valence and conduction bands of titania consist of O (2p) and Ti (3d) orbitals. The pristine film shows a strong absorption of UV light with a bandgap of 2.7 eV (Figure S7, Supporting Information). It is known that the energetic photons from UV lamps predominately exhibit electronic excitation.^[^ ^17^, ^41^, ^42^ ^]^ In this scenario, band‐to‐band excitation is achieved when photons with an energy above the titania bandgap promote a charge transfer from O^2−^ to Ti^4+^ in the TiO_6_ octahedron. The excited electron and hole in the nanocrystals are deactivated by the recombination of electron‐hole pairs, producing an emission peak at 439 nm in the PL spectra (Figure 3; Figure S8, Supporting Information). However, in an amorphous structure, the excited electrons do not decay back but are localized around disordered structures,^[^ ^41^, ^44^ ^]^ forming defects of oxygen vacancy and Ti^3+^. These defects can be evidenced by an enhanced band tail in the absorption spectra (Figure S7, Supporting Information) and the strong emission peaks at 459, 484, and 513 nm in the PL spectra (Figure 3; Figure S8, Supporting Information). The sol–gel‐derived amorphous titanium oxide features the richness of hydroxyl groups, which is attributed to the Ti─OH bonds, suggested by the broad peak at ≈3400 cm^−1^ in FTIR spectra (Figure S2a, Supporting Information). Compared to the Ti‐O in titanium oxides, the dissociation energy of the Ti─OH bond is much lower and located at 289≈482 kJ.mol^−1^.^[^ ^45^ ^]^ Under UV irradiation, the energetic photons break the T─OH bonds, eliminating the H_2_O molecule to form a bridging oxygen (Ti‐O‐Ti) between two adjacent octahedral units.^[^ ^25^, ^46^ ^]^ This process drives the dehydration and densification processes and facilitates the rearrangement of the octahedral TiO_6_ units, culminating in crystallization into the anatase phase.

### 2.3 Titania Morphologies

**Figure 4:**
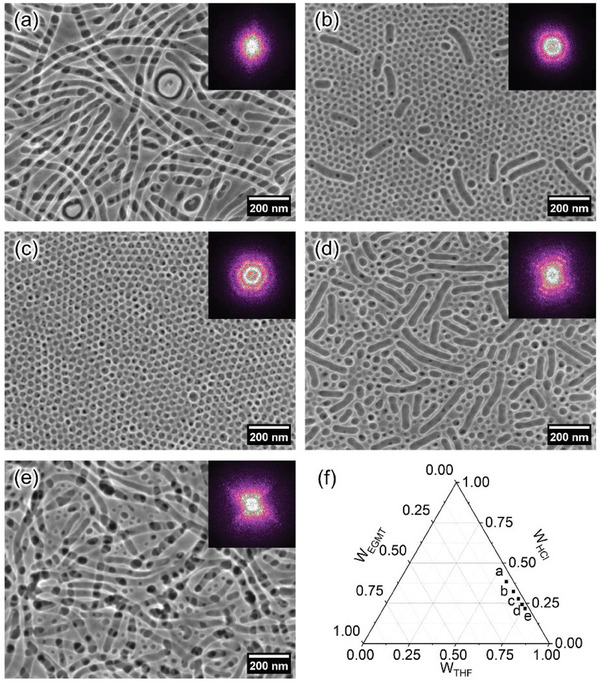
SEM images of mesoporous titania after UV irradiation with different ratios of THF and HCl. The corresponding 2D FFT patterns are inset in the upper right of SEM images. The weight ratios of THF and HCl are proportionally increased from (a) 1.5 to (e) 3.5. f) Phase diagram of these samples.

The BCP templated sol–gel synthesis is powerful and affords many possibilities of morphologies by tuning the phase separation behavior of BCP/precursor composites. The phase diagram of the BCP/precursor composites can be established by varying the fraction of precursor, BCP, and different solvents.^[^ ^47^, ^48^, ^49^, ^50^, ^51^ ^]^ This UV‐assisted low‐temperature process only removes polymer and induces crystallization without resulting in structure collapse. Here, we demonstrate that various morphologies are accessible with a tuning phase diagram. By changing the ratio of THF and HCl, cylinders, spheres, and hybrid morphologies are obtained. **Figure** **4**a–e shows the SEM images of different morphologies and their corresponding 2D fast Fourier transform (2D FFT) patterns. Figure 4f shows the phase diagram of samples in Figure 4a–e. The power spectral density (PSD) functions are extracted from the SEM images (Figure S9, Supporting Information). Figure 4a,e shows that randomly packed cylinders are obtained with the weight ratio of THF/HCl at 1.5 and 3.5. The 2D FFT images show diffuse bright patterns, indicating the broad distribution of center‐to‐center (C‐C) distance. The PSD functions of these samples feature multiple peaks with low *k*, representing large structures with 54 and 46 nm sizes for the ratios of 1.5 and 3.5, respectively. These cylinders result from the balance of interfacial tension, PEO stretching, and PS repulsion. The micelles tend to merge and form cylinders during deposition, and this coalescence is highly dependent on the local kinetics of the micelle composites.^[^ ^52^ ^]^ The morphologies transform into a hybrid of cylinders and spherical pores at the ratio of 2.0 and 3.0. With these solution compositions, the ratio of cylinders decreases significantly, with much more spherical pores appearing. The cylinders also shrink in their length. The 2D FFT image of Figure 4b shows two smeared hexagonal rings, indicating a hexagonal distribution with less ordering. A well‐ordered structure is obtained at the ratio of 2.5, as shown in Figure 4c and its 2D FFT image. The spherical pore shows a hexagonal and square distribution hybrid confirmed by a stretched hexagonal ring set in a 2D FFT image. According to its PSD profile, a peak at *k* = 0.191 nm^−1^ is observed, resulting in a C‐C distance of 33 nm. The low magnification SEM (Figure S10, Supporting Information) reveals clear contrast patterns forming various domains. These Moiré fringes are generated from stacking two similar order patterns with slight mismatching or misalignment.^[^ ^53^ ^]^ Thus, the low magnification SEM image confirms the long‐range and large area ordering of the titania films at the ratio of 2.5.

**Figure 5:**
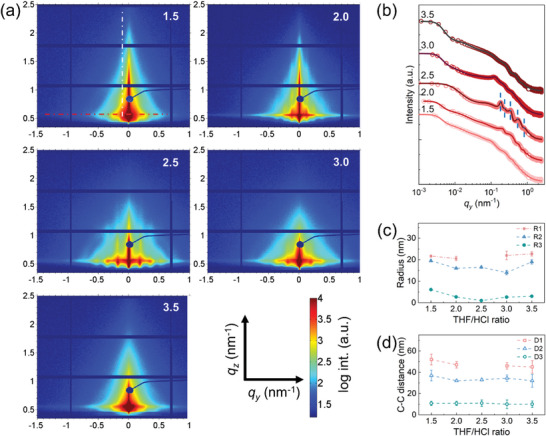
The GISAXS data and analysis of samples with different morphologies. a) 2D GISAXS data of mesoporous titania films with different ratios of THF and HCl: 1.5, 2.0, 2.5, 3.0, 3.5; the red and white dashed lines refer to horizontal and vertical line cuts respectively. b) Horizontal line cuts of the 2D GISAXS data in a log‐log style presentation. The data are plotted as hollow circles, and the modeling results based on DWBA are plotted as solid lines. The plots are shifted vertically to stack together for comparison and presentation. The blue dashed line is positioned at the Bragg peaks. The position ratio indicated by blue dashed lines is 1:$\sqrt{2}$:$\;\sqrt{4}$:$\sqrt{9}$:$\;\sqrt{20}$. The morphology information is extracted in terms of (g) radius and (h) C‐C distance from the horizontal line cut modeling. The red squares, blue triangles, and green spheres refer to structures of large size, medium size, and small size.

GISAXS measurements are carried out as a complementary method to the SEM analysis to provide statistical information of surface and buried structures.^[^ ^54^, ^55^ ^]^ With a beam size of 42×20 µm^2^ and an incident angle of 0.4°, an ellipse of 2860×42 µm^2^ on the sample is illuminated by the X‐ray beam, resulting in a high statistical relevance of the obtained morphology information. The bulk information on the whole film thickness is collected as the incident angle is much larger than the critical angle of all involved materials. **Figure** **5**a shows the 2D GISAXS data of titania films with different ratios of THF/HCl. The corresponding ratios are inset at the top right of each 2D GISAXS data. Distinct scattering patterns along the in‐plane and out‐of‐plane directions at different ratios are observed in the 2D GISAXS data. The scattering patterns are associated with the electron density difference and volume fraction of titania structures and empty pores. At the ratios of 1.5 and 3.5, strong diffuse scattering signals are observed with four sets of pine tree‐like signals embedded together, indicating randomly packed cylinders. As the ratios turn to 2.0 and 3.0, Bragg peaks appear with ring‐like signals from the form factor of spheres.^[^ ^56^ ^]^ The Bragg peaks become more prominent with several high‐order peaks at the ratio of 2.5. The shape and density changes in 2D GISAXS data give us a direct impression of structural changes over tuning the ratio of THF/HCl. The 2D GISAXS data confirm that the morphologies observed in SEM images are at the surface and extend to the whole film. A quantitative analysis is performed by extracting the vertical and horizontal line cuts from the 2D GISAXS data, as shown by the white and red dashed lines in Figure 5a.

The vertical line cuts are plotted as a function of exit angles (Figure S11, Supporting Information). The scattering signal is greatly enhanced when the exit angle of scattered X‐ray is close to the critical angle of materials due to the Vineyard effect.^[^ ^57^, ^58^ ^]^ This local intensity maximum, the so‐called Yoneda peak, is associated with the critical angle of the materials. The Yoneda peaks of the mesoporous structures are highly morphology‐dependent since different porosities result in different average electron densities and, accordingly, different critical angles of the mesoporous titania films. The process of porosity determination based on GISAXS is described in detail in the Supporting Information. The porosities of these films are estimated to be 36.58%, 30.89%, 30.89%, 47.23%, and 44.66% for the THF/HCl ratios of 1.5, 2.0, 2.5, 3.0, and 3.5, respectively. This finding is consistent with the results from a previous study that ordered structures have a lower porosity than foam‐like or worm‐like mesostructures.^[^ ^59^ ^]^

The horizontal line cuts of the 2D GISAXS data are extracted at the Yoneda peak position to derive in‐plane information (Figure 5b). All line cuts show differently pronounced peaks in the *q*_y_ range of 0.06–1 nm^−1^, indicating different extents of ordering in size or packing of the mesoporous titania nanostructure. These cuts are further modeled within the Distorted Wave Born Approximation (DWBA) and local monodisperse approximation (LMA) to provide in‐depth insights into the mesoporous structures. DWBA accounts for the multiple reflection/refraction effects in GISAXS data modeling. LMA is adapted to simplify the modeling based on an assumption of the same shape and size in local domains. More details of GISAXS modeling are shown in the Supporting Information. According to the SEM images, a cylindrical model is used for data from the ratios of 1.5 and 3.5, and the spherical model is used for data at the ratio of 2.5, while a hybrid of two models is adopted for data modeling from the ratio of 2.0 and 3.0. The domain radius (R) and domain C‐C distance (D) can be resolved by the modeling. Three sets of characteristic structures are applied for modeling the structures except for the structure at the ratio of 2.5, in which two structures are used since there are no large structures from cylinders. Footnotes 1, 2, and 3 represent the large, medium, and small structures, respectively.

For samples with ratios of 1.5 and 3.5, their horizontal profiles show several broad peaks resulting from stacking pine tree‐like signals. These broad peaks result from a form factor with a narrow distribution, namely scattering objects of similar size and shape. This finding is consistent with the results from the SEM images. For these samples, cylinders with similar radii are dominating the structure. The modeling results of these two films suggest that large and medium‐sized structures have similar radii at ≈21 nm but different C‐C distances. The D_1_ and D_2_ values are 52 and 37 nm for the ratio of 1.5 and 45 and 32 nm for the ratio of 3.5. This also holds for the sample with a ratio of 3.0. At a ratio of 2.0 or 2.5, ordered structures are formed. In these circumstances, the structure factor dominates the scattering profile with the modulation from the form factor, which means that the peaks in the horizontal line cuts are the results of the ordered spatial distribution of the pores. According to the modeling, the pore has a size of 15 nm (R_2_) with a C‐C distance of 33 nm (D_2_). In addition, the ratio of peak positions gives information on the packing mode of the pores. The positions of Bragg peaks are highlighted with blued dashed lines in Figure 5b. Body‐centered cubic (BCC) packing gives a peak position ratio of 1:$\sqrt{2}$:$\;\sqrt{4}$:$\;\sqrt{9}$:$\;\sqrt{20}$, etc. in the horizontal line profile at the Yoneda band as summarized in Table S3 (Supporting Information).^[^ ^60^ ^]^ According to the position ratios in Figure 5b, a BCC packing is determined for samples with ratios of 2.5. The (110) of BCC packing gives a distorted hexagonal distribution as shown in Figure 4c for the ratios of 2.5. The distorted 2D FFT pattern in Figure 4c confirms this distribution. Small areas of square‐like packing originate from the (100) of the BCC lattice.

### 2.4 Optical Properties with Different Morphologies

**Figure 6:**
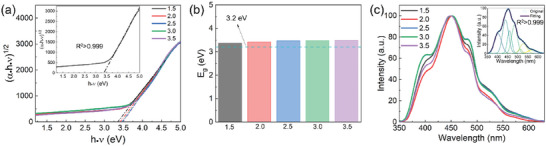
Optoelectronic properties of samples with different morphologies. a) Tauc plots from UV–vis absorption spectra with the indirect allowed transition for titania. Dashed lines indicate the corresponding linear fits of Tauc plots. The top left inset refers to an example of the linear fit. b) Bandgap values of different samples extracted from Tauc plots. c) PL spectra are recorded in the range of 350–670 nm; the deconvolution of the PL spectra is conducted with Gaussian fits, as shown in the inset.

The optoelectronic properties of samples with different morphologies are obtained by PL and UV–vis measurements. The Tauc plots of samples extracted from UV–vis absorption spectra are shown in **Figure** **6**a. According to the Tauc plots, these films show similar absorption behavior. The indirect bandgaps are determined by linear fits of the Tauc plots (Figure 6a inset). From fits of the Tauc plots, the indirect bandgaps are ≈3.4 eV (Figure 6b), close to the theoretical bandgap of 3.2 eV of anatase. The deviation of the bandgap from the theoretical value is associated with the defects in the crystal lattice, as indicated by the Urbach tail in Figure 6a. The defect states of titania are investigated by PL emission spectra excited at 3.87 eV (320 nm). As shown in Figure 6c, all these films have very similar PL spectra but show slightly different intensities of defect states, which result from the processing. The deconvolution of the PL spectra is carried out by Gaussian fits (Figure 6c inset; Figure S6, Supporting Information), and the attributions of different defects are summarized in Table S2 (Supporting Information). The emission peaks at 400 and 439 nm are attributed to band edge emission of self‐trapped excitons and the recombination of electron‐hole pairs, respectively. The peaks at 459, 484, and 513 nm are attributed to different processes, but all are related to oxygen vacancies. The 528 and 572 nm peaks are associated with one‐trapped electrons to the valence band of TiO_2_ and deep trap states due to lattice disorder/oxygen vacancies. The PL deconvolution results indicate that the primary defects in the samples are natural oxygen vacancies, which are widely present in titania.^[^ ^61^, ^62^, ^63^, ^64^ ^]^

### 2.5 General Applicability

**Figure 7:**
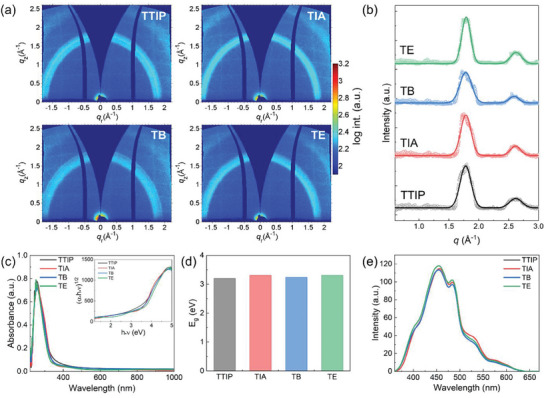
The general applicability of UV irradiation on different titanium precursors. a) 2D GIWAXS data of titania films prepared from different titanium precursors after UV irradiation; the top right insets refer to the names of the precursors. b) *Pseudo*‐XRD data extracted from 2D GIWAXS data for different titania films are plotted with hollow circles, and the Gaussian fit results are plotted as solid lines. All the data and fits are shifted vertically for presentation. c) UV–vis absorption data of titania films from different precursors; the top right inset refers to Tauc plots from UV–vis absorption spectra with the indirect allowed transition for titania. d) Bandgap of different samples extracted from Tauc plots. e) PL spectra of different titania films.

The introduced low‐temperature fabrication mediated by UV irradiation can be extended to different titanium precursors beyond EGMT. Titanium alkoxides are the most commonly used titanium sources in the sol–gel process due to their commercial availability and established application in the synthesis of titania. As an example, four commercial representative titanium alkoxides, namely titanium tetraisopropoxide (TTIP), titanium diisopropoxide bis(acetylacetonate) (TIA), titanium butoxide (TB), and titanium ethoxide (TE), are selected to demonstrate the general applicability of our synthesis route. The differences between these precursors are summarized in the Table S4 (Supporting Information). The different structures of these precursors influence their reactivities, hydrolysis‐condensation rates, and the properties of the resulting nanoparticles. For example, slower hydrolysis‐condensation typically yields smaller nanoparticles, which can affect interactions with polymer templates, leading to variations in mesoporous structures. The hydrolysis‐condensation process was deliberately controlled by adding HCl and extending reaction times, minimizing precursor‐induced differences, and producing amorphous nanoparticles of similar size for co‐assembly to demonstrate that the UV‐assisted fabrication method is independent of precursor chemistry. The SEM images (Figure S12, Supporting Information) reveal similar ordered morphologies for the films prepared from different precursors. **Figure** **7**a shows the 2D GIWAXS data of UV irradiated titania films from TTIP, TIA, TB, and TE. Obvious signals from the (101) reflex of anatase are observed in the 2D GIWAXS data. The *pseudo*‐XRD data (Figure 7b) confirms the formation of anatase with characteristic peaks located at *q* = 1.79 and 2.66 Å^−1^, corresponding to (101) and (004) signals from anatase. The anatase has lattice constants *a = b* = 3.78 Å and *c =* 9.44 Å with a tetragonal structure. The optoelectronic properties of mesoporous titania films from different precursors are further examined by UV–vis measurements and PL measurements. Figure 7c–e are the UV–vis absorption spectra, bandgaps extracted from absorption spectra, and the PL spectra of different titania films. These films show similar absorption behavior with similar absorption peaks at 270 nm (Figure 7c), similar bandgaps at ≈3.3 eV, and similar PL spectra. The similarity of the optoelectronic properties indicates the general applicability of UV irradiation. The prepared mesoporous titania films can potentially be used for different applications, such as photocatalysis, as demonstrated by the photocatalytic degradation of methyl red (Figure S13, Supporting Information).

## 3 Conclusion

We demonstrate a versatile strategy of synthesizing mesoporous titania films in low‐temperature conditions to be a novel route in titania synthesis, replacing the commonly applied calcination process. UV irradiation is proven to remove the polymer templates and induce titania crystallization. With increasing irradiation time, the titania crystal size and relative degree of crystallization increase and finally stay stable. These evolutions are also verified by PL measurements. The crystal size and relative degree of crystallization of samples from UV irradiation are comparable to those from classical calcination routes but without structure sacrifice via pattern collapse. Using BCP templated sol–gel synthesis, various morphologies, including cylinders, spheres, and their hybrids, can be obtained as revealed by SEM and GISAXS data. Films with different morphologies show similar optoelectronic properties. Notably, the introduced UV irradiation‐mediated low‐temperature fabrication is versatile and applicable for different titanium precursors, as confirmed by four commercial titania precursors. This general applicability of the UV‐assisted low‐temperature fabrication holds promise for applications on other mesoporous metal oxides.

## 4 Experimental Section

### Materials

The ethylene glycol‐modified titanate (EGMT) was synthesized via a polyol process from the literature.^[^ ^65^, ^66^ ^]^ The EGMT is a white powder with a crystalline structure, which was confirmed by XRD measurement (Figure S14, Supporting Information). Tetrahydrofuran (THF, anhydrous, ≥99.9%), hydrochloric acid (HCl, 37%), titanium tetraisopropoxide (TTIP, 99.999%), titanium diisopropoxide bis(acetylacetonate) (TIA, 75 wt.% in isopropanol), titanium butoxide (TB, 97%), titanium ethoxide (TE, 97%), and methyl red (ACS reagent) were purchased from Sigma–Aldrich, Germany. The BCP polystyrene‐block‐polyethylene oxide (PS‐b‐PEO, with polydispersity index: 1.02) was purchased from Polymer Source Inc., Canada. The average molecular weights of the PS and PEO blocks were 20500 and 8000 g mol^−1^, respectively. All chemicals and materials were directly used as obtained. The ITO‐coated PET sheet (thickness of 0.25 mm) was obtained from Sigma–Aldrich, Germany.

### Preparation of Mesoporous Titania Films at Low Temperatures

The synthesis of mesoporous titania films was based on an amphiphilic BCP templated sol–gel method. The PS‐*b*‐PEO served as a soft template, and titania precursors interacted with hydrophilic block PEO selectively via non‐covalent bonds. In a typical synthesis, 12 mg PS‐*b*‐PEO was dissolved in 1.4 mL THF. After entirely dissolving the polymer, a desired amount of HCl solution was added to the above solution with stirring for 20 min, followed by an addition of 0.3 mmol EGMT precursor. The solution was then transferred into a water bath and stirred for 3 h at 65 °C to ensure the complete hydrolysis of the precursors. The weight ratio of THF and HCl was varied to tailor the morphology of titania films. For the four commercial titanium precursors, the only variance of the protocol was the ratio of THF and HCl, which was kept at 10 according to the literature to obtain mesoporous structures.^[^ ^48^ ^]^

The final sol–gel solutions were spin‐coated on precleaned substrates with a spin coating speed of 1800 rpm for the 80 s. Silicon (Si) substrates were used for morphology characterization and X‐ray scattering measurements, and glass substrates were used for optoelectronic characterization. The obtained thin films were composites of polymer and amorphous titania. The deposited thin films were then irradiated under UV to remove the polymer template and induce crystallization of titania. The UV lamp (HTC 400‐241, SUPRATEC) had a UVA radiant power of 82 W and a UVB radiant power of 12 W. The samples were put at a distance of 10 cm under the lamp for the irradiation treatment in an ambient atmosphere. Due to the heat emission from the lamp, the temperature of the chamber was at 60–70 °C during UV irradiation. Calcination at 400 °C for 3 h was used to produce reference samples. Afterward, the mesoporous titania films were obtained and investigated. For the demonstration of the compatibility on thermosensitive flexible substrates, ITO‐coated PET was used with slot‐die coating. The parameters for the slot die coating were the same as in the previous report.^[^ ^49^ ^]^ The photocatalysis was carried out with mesoporous titania films. The concentration of the methyl red solution was set to 0.04 mg mL^−1^. To establish the adsorption equilibrium between methyl red and titania, the titania films were immersed in the dye solution, shaking for 6 h in the dark. The solution after the adsorption was the original solution. The dye solutions with titania films were put under UV light (365 nm) illumination for another 6 h of photocatalytic degradation at room temperature.

### Characterizations

SEM (Zeiss Gemini NVision 40) measurements were performed to investigate the sample topology with an accelerating voltage of 5.0 kV and a working distance of 5 mm. The image analysis of SEM images was performed within Gwyddion to extract 2D fast Fourier transform (2D FFT) and power spectral density (PSD) functions.^[^ ^67^ ^]^ GISAXS measurements were conducted at the P03/MiNAXS beamline of the PETRA III storage ring at Deutsches Elektronen‐Synchrotron (DESY, Hamburg, Germany).^[^ ^68^ ^]^ The X‐ray energy was set to 11.81 keV, and the sample‐to‐detector distance (SDD) was calibrated to 3090 mm with standard reference sample silver behenate. The scattering signal was recorded with a Pilatus 2 m detector (Dectris Ltd., pixel dimension 172 µm × 172 µm) with an incident angle of 0.4°. The momentum transfer *q_y_* and *q_z_* were the in‐plane and out‐of‐plane scattering vectors. The GISAXS data were analyzed using the software DPDAK.^[^ ^47^ ^]^ GIWAXS measurements were performed with an in‐house Ganesha 300XL SAXS‐WAXS instrument with an 8.047 keV Cu Kα X‐ray source. The scattering signal was recorded with a Pilatus 300k detector (Dectris Ltd.) at an SDD of 95.67 mm and an incident angle of 0.4°. The GIWAXS data were processed by the GIXSGUI software.^[^ ^69^ ^]^ More details of X‐ray scattering data analysis are discussed in the Supporting Information.

UV–vis measurements were performed with a spectrophotometer (Lambda 35, PerkinElmer). PL measurements were carried out with a PerkinElmer LS 55 luminescence spectrometer. For emission scans, the excitation wavelength was fixed and recorded after the excitation wavelength. The excitation scans instead fixed the emission recording wavelength and varied the excitation wavelength. For all PL measurements, high‐pass emission filters with different wavelengths were used to minimize the influence of second‐order scattering. FTIR measurements were conducted with an Equinox 55 (Bruker Optik GmbH, Rosenheim, Germany) spectrometer.

## Conflict of Interest

The authors declare no conflict of interest.

## Supporting information



Supporting Information

## Data Availability

The data that support the findings of this study are available from the corresponding author upon reasonable request.
