# Supplementary material for: UV Irradiation as a Versatile Low‐Temperature Strategy for Fabricating Templated Mesoporous Titania Films
Source: Small. 2024 Dec 17;21(5):2409856. doi: 10.1002/smll.202409856 (PMC11798364; doi:10.1002/smll.202409856)
Supplement: Supplementary file 1 — Supporting Information [file SMLL-21-2409856-s001.pdf]

NANO · MICRO
small

Supporting Information

for *Small*, DOI 10.1002/smll.202409856

UV Irradiation as a Versatile Low-Temperature Strategy for Fabricating Templated Mesoporous Titania Films

*Guangjiu Pan, Shanshan Yin, Linus F. Huber, Zerui Li, Ting Tian, Lukas V. Spanier, Huaying Zhong, Tianfu Guan, Caroline R. Ehgartner, Nicola Hüsing, Matthias Schwartzkopf, Stephan V. Roth and Peter Müller-Buschbaum**

Supporting Information

UV irradiation as a versatile low-temperature strategy for fabricating templated mesoporous titania films

*Guangjiu Pan, Shanshan Yin, Linus F. Huber, Zerui Li, Ting Tian, Lukas V. Spanier, Huaying Zhong, Tianfu Guan, Caroline R. Ehgartner, Nicola Hüsing, Matthias Schwartzkopf, Stephan V. Roth, Peter Müller-Buschbaum**

G. Pan, S. Yin, L. F. Huber, Z. Li, T. Tian, L. V. Spanier, H. Zhong, T. Guan, Peter Müller-Buschbaum
Chair for Functional Materials, Department of Physics, TUM School of Natural Sciences, Technical University of Munich, Garching, 85748, Germany
Email: muellerb@ph.tum.de
S. Yin
School of Mathematics and Physics, Jiangsu University of Technology, Changzhou, 213001, China
C. R. Ehgartner, N. Hüsing
Chemistry and Physics of Materials, Paris-Lodron University Salzburg, Jakob-Haringer Straße 2a, Salzburg, 5020, Austria
M. Schwartzkopf, S. V. Roth
Deutsches Elektronen-Synchrotron DESY, Notkestraße 85, Hamburg, 22607, Germany
S. V. Roth
Division of Coating Technology, KTH Royal Institute of Technology, Teknikringen 48, Stockholm, 100 44, Sweden

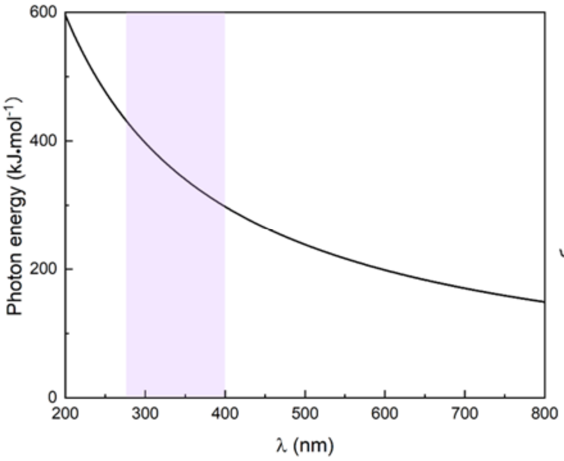

Bond	Bond energy ($kJ.mol^{-1}$)	λ (nm)
C-H	413	290
C-C	347	345
C-O	358	334

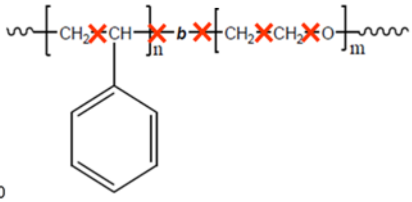

Figure S1. (left) Photon energy diagram as a function of wavelength with the UVA and UVB ranges highlighted by a purple rectangle; (upper right) table of bond information, including the bonds in the backbone, their dissociation energy, and corresponding wavelength; and (lower right) sketch of PS-*b*-PEO and possible breaking points as highlighted by the red crosses.

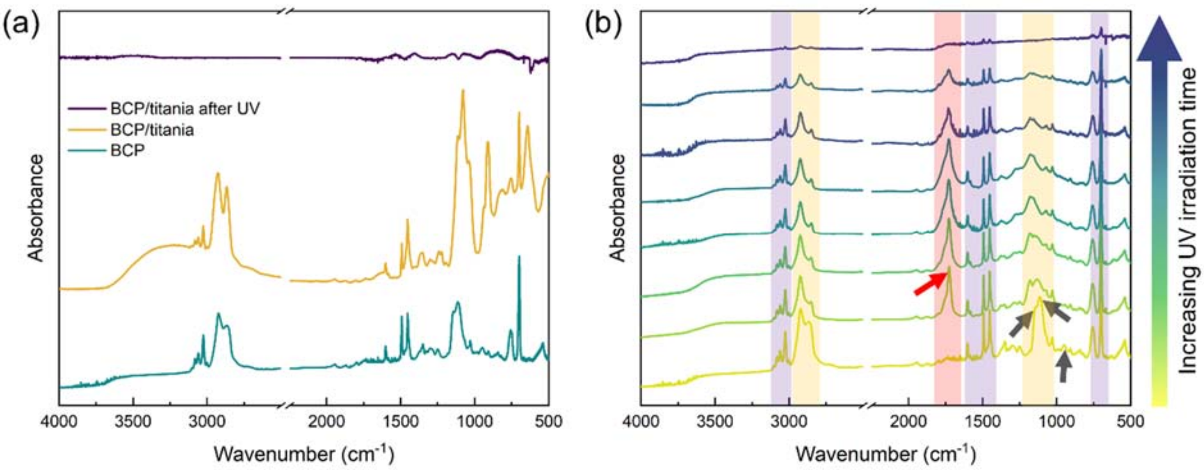

Figure S2. (a) FTIR spectra of BCP, titania/BCP composite films before and after the UV irradiation. The spectra are shifted vertically. The clean spectrum of the film after UV irradiation indicates the removal of the polymer template. The broad peak at around 3400 cm^{-1} corresponds to the hydroxyl group, which is from amorphous titania. (b) FTIR spectra of pure BCP film at different times of UV illumination. The purple-shaded rectangles indicate the contribution of the aromatic ring. The yellow rectangles highlight the disappearance, and the red rectangle shows the appearance of signals. After UV irradiation, the signals from C-O-C disappear, and signals from C=O appear as indicated with black and red arrows, respectively. This evolution verifies the breaking of C-O-C and the involvement of C=O during the photolysis of PEO and PS.

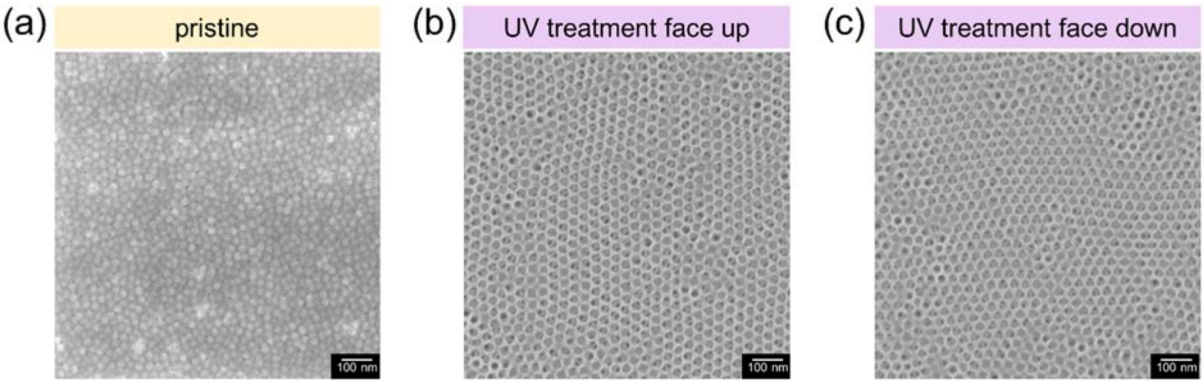

Figure S3. SEM images of (a) pristine film and films after UV treatment with the sample surface, (b) face up to the UV light for 4 h, and (c) face down for 12 h. The nanospheres in the pristine film come from the block copolymer micelles. (b and c) Both films show a mesoporous morphology, indicating that the block copolymer micelles were removed. The removal of polymer in the indirect interaction with UV light confirms the role of UV-induced radicals.

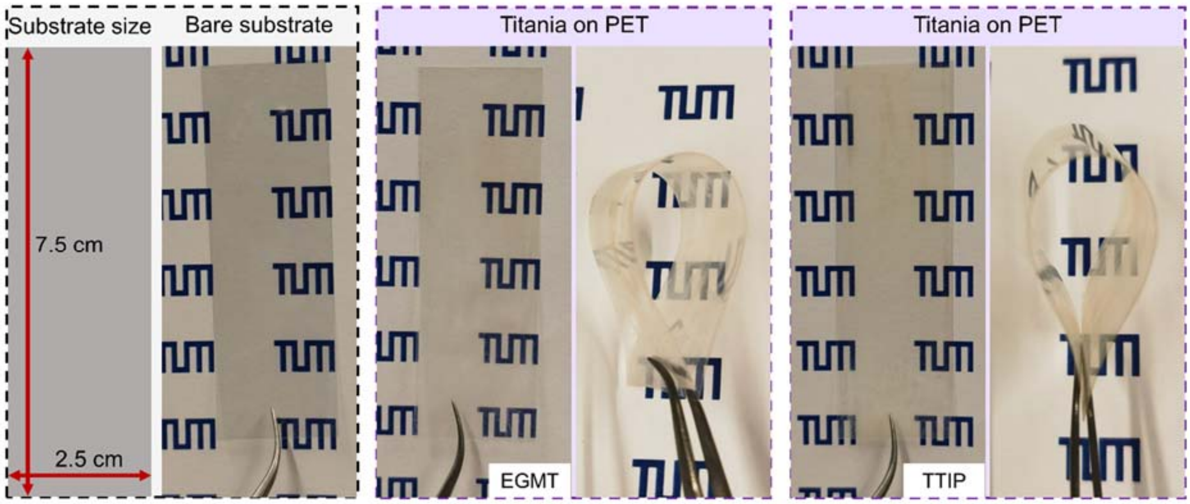

Figure S4. Digital photos of the bare substrate and two titania films from different precursors on PET substrate after 54 h UV irradiation. The substrates were cut into width × length of 2.5 cm × 7.5 cm to fit the stage size of the used slot-die coater.

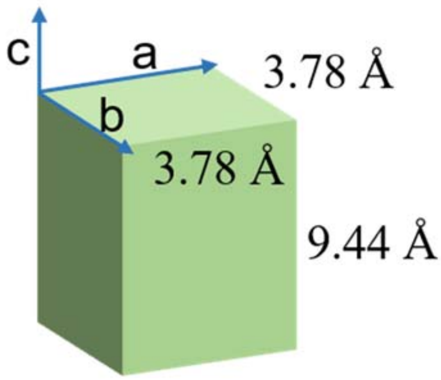

Figure S5. Sketch of the crystal structure of anatase. With a tetragonal structure, the crystal has lattice constants of a=b=3.78 Å and c=9.44 Å.

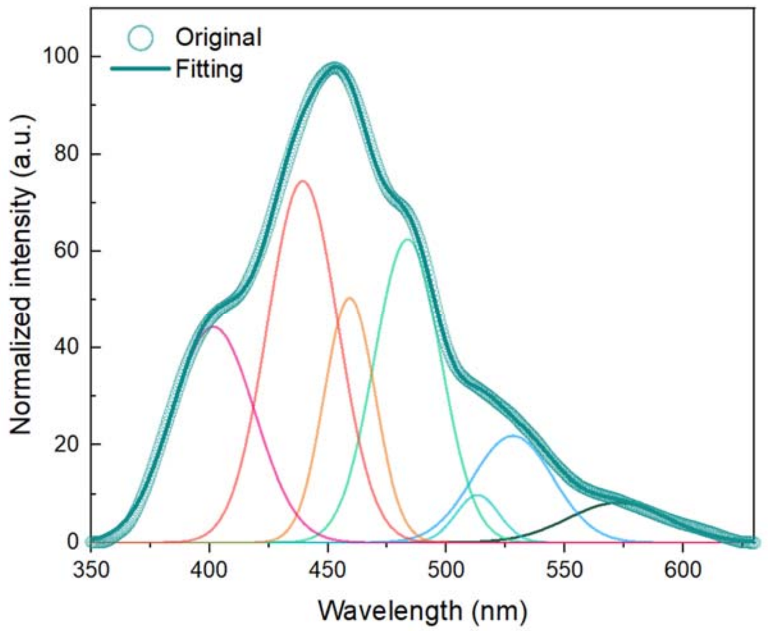

Figure S6. Deconvolution of PL spectrum. The emission peak is Gaussian-fitted. The data are plotted with hollow circles, and the deconvoluted spectra are plotted with solid lines. For clarity, the cumulative fit profile (green solid line) is superimposed on the experimental data.

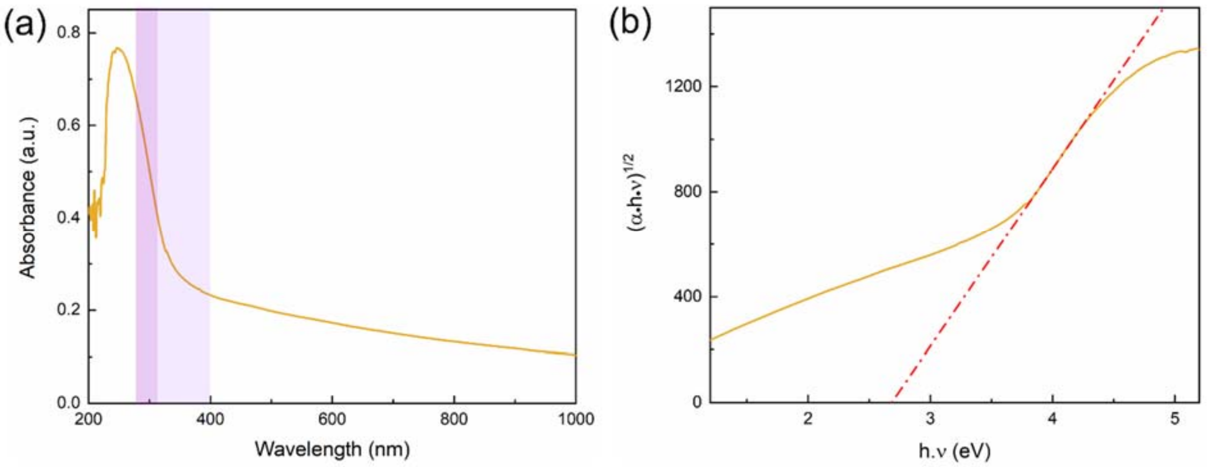

Figure S7. (a) UV-Vis absorption data of titania film before UV irradiation; the dark and light purple rectangles highlight the UVB and UVC wavelength ranges. (b) Tauc plot from UV-Vis absorption spectra with the indirect allowed transition for titania; the dashed red line refers to the linear fit, indicating a band gap of 2.7 eV.

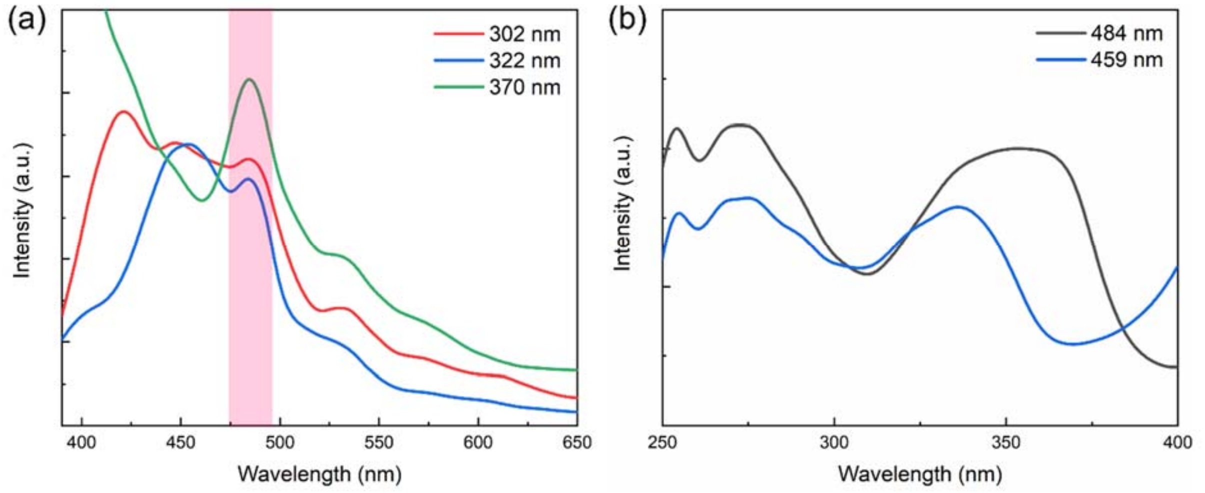

Figure S8. (a) Emission spectra of a pristine titania film at different excitation wavelengths of 302, 322, and 370 nm. The excitation wavelengths are chosen to represent the UV lamp radiation peaks. The emission peak attributed to the charge-transfer process from Ti^{3+} to the nearby oxygen anion in a TiO_6 octahedron is highlighted with a red rectangle. (b) Excitation scan recorded with different emission peaks of 484 and 459 nm. These two emission peaks are all associated with oxygen defects. The excitation spectra show strong emission after absorbing UVA photons.

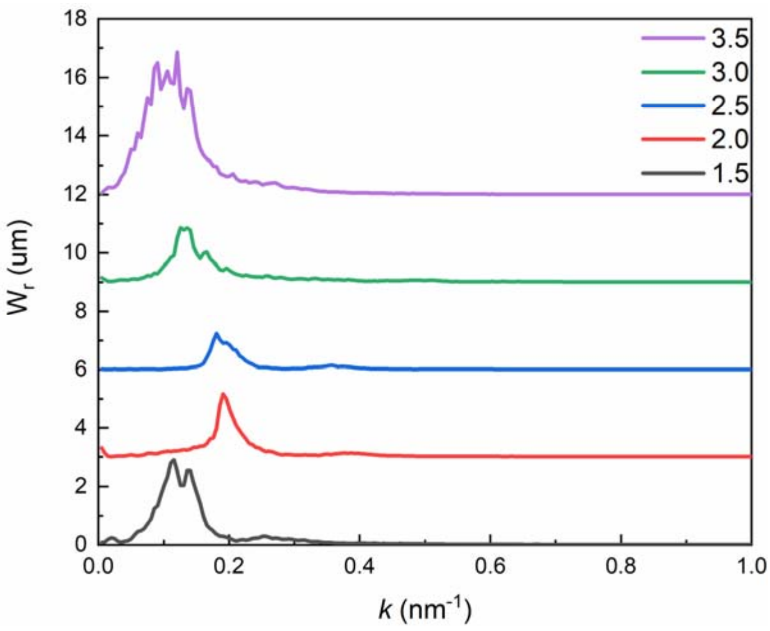

Figure S9. Power spectral density (PSD) functions extracted from SEM images. The plots are shifted vertically. The peaks in the spectra come from the Fourier transform of the structures, indicating ordered characteristic sizes. By using $d = 2\pi/k$, the characteristic interdomain distance d is determined. The characteristic d values (corresponding to the highest peaks) for titania films with different THF/HCl ratios are determined to be 54 nm, 33 nm, 34 nm, 46 nm, and 60 nm, respectively.

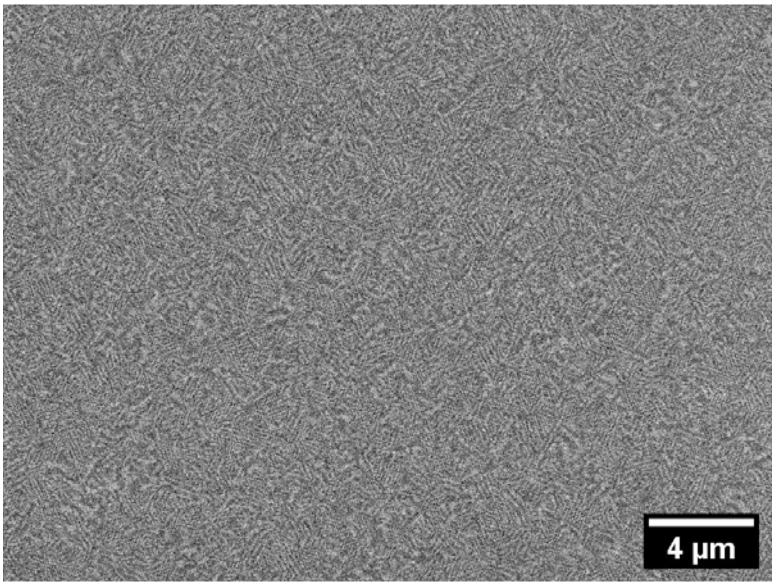

Figure S10. SEM image of the mesoporous titania surface at a magnification of 4000. The domain size of the ordered structure is larger than 1 μm. The moiré fringes are generated from stacking two similar order patterns with slight mismatching or misalignment. The domains with low contrast do not indicate disorder but indicate Moiré conditions unfulfilled (relative rotation > 20°).

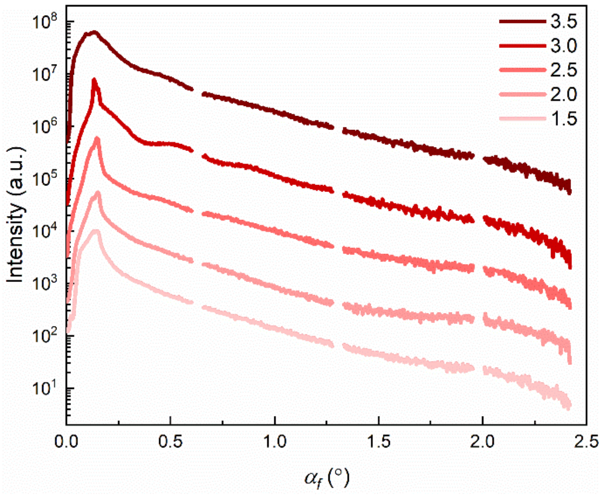

Figure S11. Vertical line cuts extracted from the 2D GISAXS data at q_y= -0.1 nm^{-1}. The ratios of THF and HCl are 1.5, 2.0, 2.5, 3.0, and 3.5, respectively. The plots are shifted vertically. The gaps in the data come from the detector gaps. The sample horizon is at α_f=0 ° and the local maximum at α_f ≈0.15 ° corresponds to the Yoneda peak of the mesoporous titania.

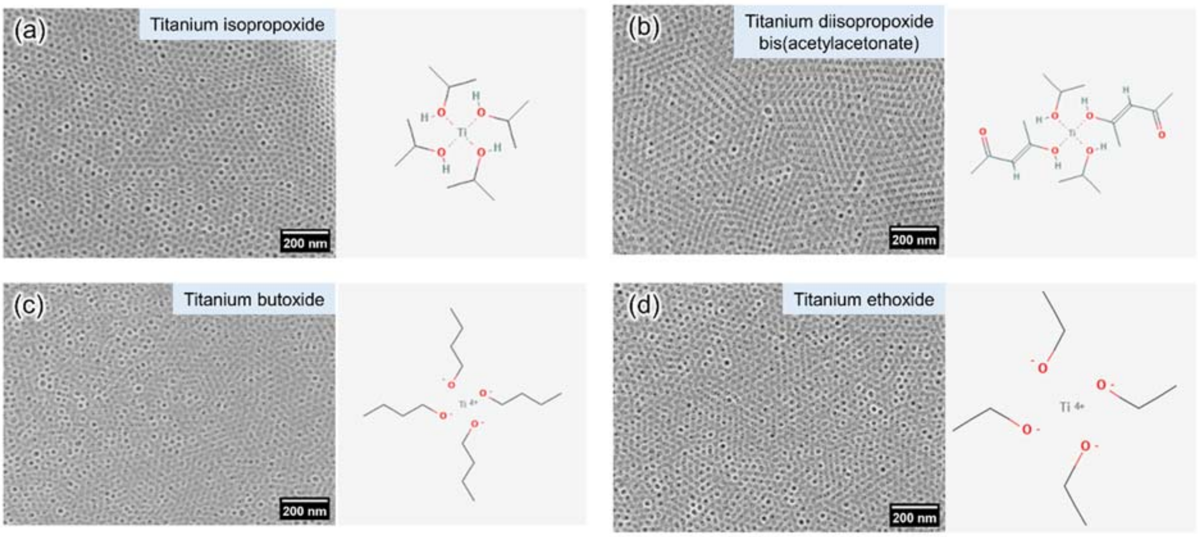

Figure S12. Thin film morphologies from different precursors and their corresponding chemical structures: (a) titanium isopropoxide, (b) titanium diisopropoxide bis(acetylacetonate), (c) titanium butoxide, and (d) titanium ethoxide. The left side shows an SEM image of the thin film morphology from these different precursors, and the right side of every figure shows the corresponding chemical structure of the precursor.

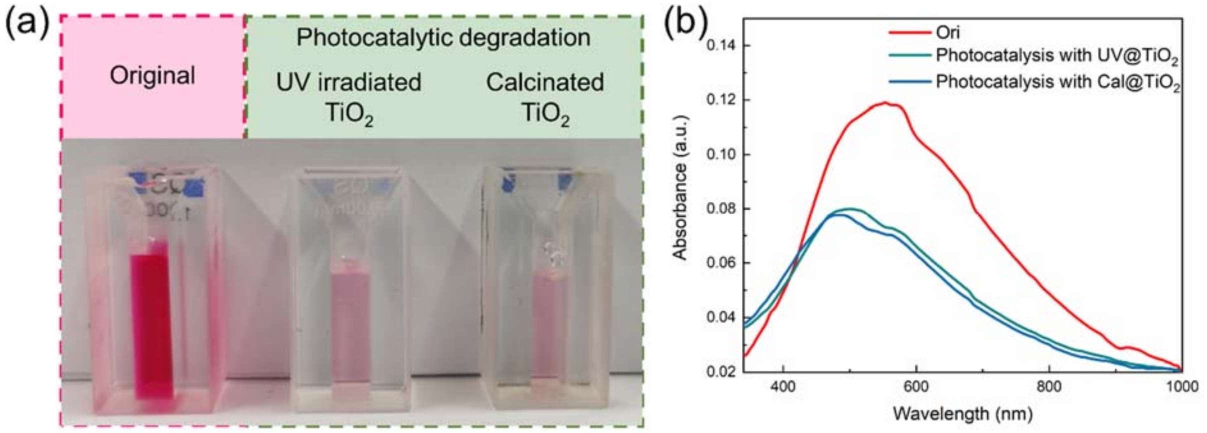

Figure S13. Photocatalytic degradation of methyl red. (a) Photographs of the methyl red solution before and after photocatalysis of 6 h with titania films and (b) their corresponding absorption spectra. The original concentration of methyl red in solution is 0.04 mg/ml with pH=2. UV-irradiated or calcinated TiO_2 mesoporous films are used for photocatalysis.

Table S1. Comparison of energy consumption between UV irradiation and calcination

	Parameters	Energy (Joule)
calcination	400 ℃, 3h	7.69×10^{9}
UV irradiation	UV irradiation power 94 W, 80h	2.71×10^{7}
	UV irradiation power 94 W, 80h, including heat produced by the lamp (70 ℃)	4.00×10^{7}

Table S2. PL deconvolution results

Location (nm)	Attribution
400	band-edge emission of self-trapped excitons
439	recombination of electron-hole pairs
459	oxygen defect-related emission peaks
484	the charge-transfer process from Ti^{3+} to the nearby oxygen anion in a TiO_6 octahedron, associated with oxygen defects
513	oxygen vacancies associated with Ti^{3+} in anatase TiO_2
528	one-trapped electron to the valence band of TiO_2
572	deep trap states due to oxygen vacancies and lattice disorder

Table S3. Summary of the theoretical peak positions for BCC packing

Peak	q_y position[#]	Position ratio	hkl	m[*]	Intensity
1	8.86	1	$1\bar{1}0$	12	48
2	12.56	$\sqrt{2}$	002	6	24
3	15.39	$\sqrt{3}$	$1\bar{1}2$	24	96
4	17.78	$\sqrt{4}$	$2\bar{2}0$	12	48
5	21.78	$\sqrt{6}$	$2\bar{2}2$	8	32
6	25.16	$\sqrt{8}$	004	6	24
7	26.64	$\sqrt{9}$	$3\bar{3}0$	12	48
8	29.48	$\sqrt{11}$	$3\bar{3}2$	24	96
9	30.78	$\sqrt{12}$	$2\bar{2}4$	24	96
10	35.55	$\sqrt{16}$	$4\bar{4}0$	12	48
11	36.63	$\sqrt{17}$	$3\bar{3}4$	24	96
12	37.71	$\sqrt{18}$	$4\bar{4}2$	24	96
13	38.74	$\sqrt{19}$	$1\bar{1}6$	24	96
14	40.72	$\sqrt{20}$	620	48	192

[#] For demonstration, the lattice parameters of BCC packing are set to a=b=c=1 without units, and thus the q positions are figures without units;

[*] m: peak multiplicities.

Table S4. Properties of different titanium alkoxides

Name	Titanium butoxide	Titanium isopropoxide	Titanium ethoxide	Titanium diisopropoxide bis(acetylacetonate)
Coordination environment[#]	octahedra	tetrahedron	octahedra	octahedron
Coordination number	5	4	5	6
Form	liquid	liquid	liquid	liquid
Hydrolysis rate (comparable)	Fast	Fast	Fast	Medium

The coordination environment is different even though the valence of the Ti atom is the same. The coordination environment and number refer to the status of the liquid precursor. Titanium alkoxides exist in trimers or multimers in a liquid state.

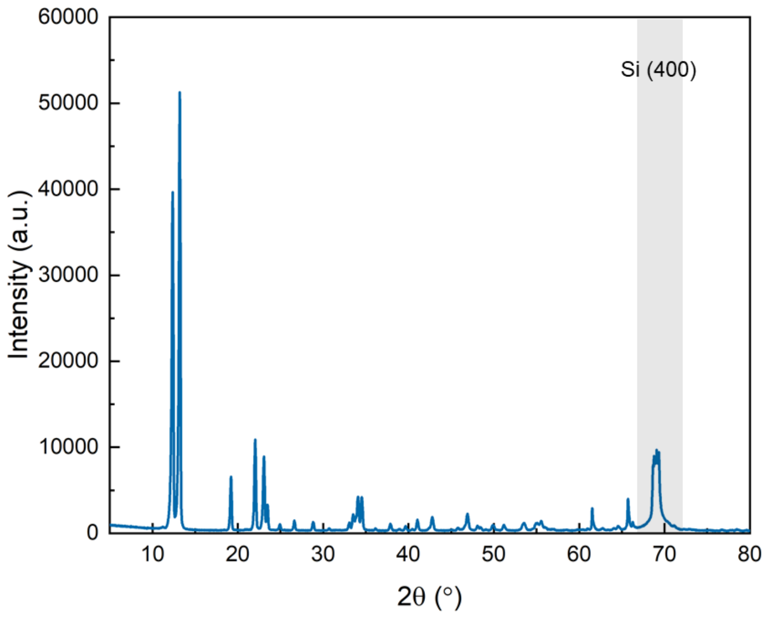

Figure S14. XRD pattern of EGMT. The grey rectangle shaded peaks are Si(400) from the Si wafer substrate.

GIWAXS data analysis

The raw GIWAXS data are corrected for missing wedges and then presented as a function of scattering vector q_r and q_z. The q_r and q_z denote the momentum transfer of in-plane and out-of-plane directions. The scattering vector coordinates are given by

$$q=\begin{pmatrix} q_x \\ q_y \\ q_z \end{pmatrix}=\frac{2\pi}{\lambda}\begin{pmatrix} \cos(\alpha_f)\cos(2\theta)-\cos(\alpha_i) \\ \cos(\alpha_f)\sin(2\theta) \\ \sin(\alpha_f)+\sin(\alpha_i) \end{pmatrix}$$

where α_i is the incident angle; α_f and 2θ are the exit angles of out-of-plane and in-plane directions, respectively. Compared to GISAXS, the q_x component cannot be ignored in the GIWAXS regime. Therefore, the in-plane scattering vector q_r is defined by

$$q_r=\sqrt{{q_x}^2+{q_y}^2}$$

a. Lattice constants

The interplane distance d_{hkl} is determined for each plane is

$$d_{hkl}=\frac{2\pi}{q}$$

where d_{hkl} is the d-spacing for a specific plane with Miller Indices hkl, and q is the corresponding peak position in reciprocal space. For a tetragonal-like anatase ($I4_1/amd$), the d_{hkl} is then related to lattice constants *a, b, c* by

$$\frac{1}{{d_{hkl}}^2}=\frac{h^2+k^2}{a^2}+\frac{l^2}{c^2}$$

Then the q_{hkl} is rewritten with lattice constants as follows

$$q_{hkl}=2\pi\sqrt{\frac{h^2+k^2}{a^2}+\frac{l^2}{c^2}}$$

By solving this formula, lattice constants a and c can be obtained.

b. Crystallinity size

The Scherrer equation was used to calculate the minimum crystallite size (more precisely, coherence length). The Scherrer analysis assumes that finite crystallite size is the only contribution to peak with a Gaussian function. The crystal size *D* is related to the peak width by

$$D_{hkl}=\frac{2\pi K}{\Delta q}$$

where *K* is the shape factor and Δq is the full width at half-maximum (FWHM) of a peak. In our case, spherical crystallites are assumed, thus resulting in a shape factor of 0.89. The experimental peak width is a wrap-up of the intrinsic peak width from the sample, instrumental broadening, monochromatic resolution, and GIWAXS footprint. A standard sample (LaB_6,

which has negligible sample broadening) is used to characterize the non-intrinsic broadening. The sample contribution to the broadening is then given as

$$FWHM_{samp} = \sqrt{FWHM_{exp} - FWHM_{ins}}$$

where $FWHM_{samp}$, $FWHM_{exp}$, and $FWHM_{ins}$ are $FWHM$ from sample broadening, experimental profile, and non-intrinsic broadening. The $FWHM_{ins}$ was extracted from GIWAXS data of standard sample LaB_6 measured at the same sample-to-detector distance and incident angle.

c. Relative degree of crystallinity

Crystallinity is important for many applications of metal oxides since amorphous regions provide lots of defects and traps that may be negative for applications in solar cells. The degree of crystallinity (*DoC*), the volume fraction of crystalline materials in a sample illuminated by an X-ray beam, is proportional to the integrated intensity of a diffraction peak. However, it is difficult to derive the absolute *DoC* due to the required reference samples of entirely crystalline and entirely amorphous films.

Here, we use a relative degree of crystallinity (*rDoC*) to compare the crystallinity of different samples. The integrated intensity of a pole figure is directly proportional to the *rDoC*. Thus, tube cuts (Figure S7a) of the peak at q=1.6~2.0 Å^{-1} ($\chi = -90°\sim90°$, where χ is the polar angle) were conducted to generate the distorted pole figure for (101) anatase (Figure S7b). The crystallites in our system are assumed as isotropic 2D powder. Due to the intrinsic imbalance counting of intensity at different polar angles, a weak intensity at high χ (χ>70° or <-70°) does not indicate a low amount of crystallites at these orientations (large Lorentz weight), and *vice-versa* for low χ area (small Lorentz weight). Thus, a Lorentz factor ($\sin\chi$) is introduced to correct the integrated intensity and generate the pole figure (Figure S7c) as follow

$$\mathrm{I} = 2\pi \int\int I(q,\ \chi) q^2 \sin\chi \, \mathrm{d}q \mathrm{d}\chi \sim \int I(q,\ \chi) \sin\chi \, \mathrm{d}\chi$$

The amount of crystallite in materials, also *rDoC*, is proportional to the pole figure area (Figure S7c). By calculating the integrated intensity in the pole figure, the *rDoCs* of different samples are determined.

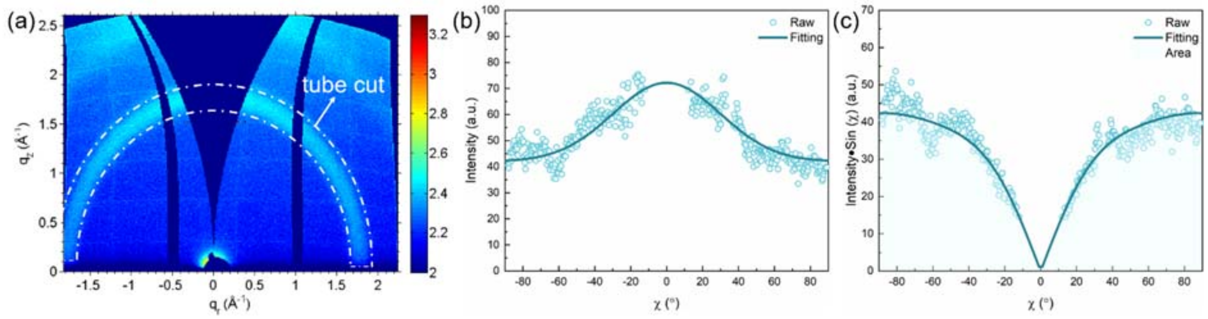

Figure S15. The process of calculating *rDoC*. (a) Corrected 2D GIWAXS data with a white dashed ring indicating the area of tube cut. (b) Tube cut result from (a) is plotted with χ; the gap centered at 0° is from the missing wedge. The data is fitted with a Gaussian peak centered at 0°. The higher intensity at low χ indicates a face-on orientation of anatase crystals. (c) Pole figure from the tube cut after correction with the Lorentz factor; the integrated area of the pole figure (shaded by light green) is proportional to the *rDoC*.

GISAXS data analysis

a. Porosity determination

The critical angle of materials is related to the electron density of thin films. Mesoporous materials have different critical angles compared to their compact, dense form. Therefore, the porosity can be calculated by comparing the critical angle of films to the theoretical values.

The scattering contrast in X-ray scattering is mainly proportional to the refractive index

$$n=1-\delta + i\beta$$

where δ is the dispersion term and of order 10^{-5} in solids, the imaginary part β is the absorption term and is usually much smaller than δ. This complex index is mainly contributed by the dispersion part. The dispersion term is closely associated with the electron density of materials. The critical scattering vector q_c is then related to *SLD* according to

$$q_c=\sqrt{16\pi SLD}$$

and the critical angle α_c can be expressed as

$$\alpha_c = arcsin\left(\frac{q_c\lambda}{4\pi}\right)$$

$$= arcsin\left(\frac{\lambda\sqrt{16\pi SLD}}{4\pi}\right)$$

$$\approx \sqrt{\frac{\lambda^2 SLD}{\pi}}$$

By taking the α_c from mesoporous samples into this equation, the *SLD* values of mesoporous (SLD_m) can then be derived. Based on the titania properties and X-ray wavelength, the theoretical *SLD* values of compact titania are summarized as follows:

	anatase
Density (gcm^{-3})	3.90
SLD ($\times 10^{-5}$ Å^{-2})	3.13

A comparison of *SLD* values of mesoporous (SLD_m) and compact (SLD_c) titania gives us a rough determination of the mesoporous of the obtained films according to

$$P=1-\frac{SLD_m}{SLD_c}$$

b. Modeling of horizontal line cuts

The probability of a scattering event is determined by the total differential scattering cross-section. Multiple scattering effects occur due to the presence of the substrate. The diffuse scattering from a surface is then given by

$$\frac{d\sigma}{d\Omega}=\frac{A\pi^2}{\lambda^2}|\Delta|^2|T_i|^2|T_f|^2P(\vec{q})$$

where A denotes the illuminated area by X-ray, $|\Delta|^2$ is the scattering contrast function, T_i and T_f are the Fresnel transmission coefficients of the incident and scattered beam. The two Fresnel transmission coefficients have a maximum at an angle close to α_c, yielding an enhanced intensity (so-called Yoneda peak). This is the reason why the horizontal cuts are performed at the Yoneda peak and the angle with local maximum intensity in vertical cuts is related to α_c. $P(\vec{q})$ is the diffuse scattering factor and given by

$$P(\vec{q}) \propto N|F(\vec{q})|^2 S(\vec{q})$$

This scattering factor holds for monodisperse objects with the number density of scattering objects N, the object form factor $F(\vec{q})$, and the structure factor $S(\vec{q})$. The form factor accounts for the shape, size and orientation of the scattering objects, while the structure factor accounts for spatial distribution, mean distance, and distance fluctuation of the objects. Due to the presence of surface, multiple scattering events need to be considered and described in the frame of distorted-wave Born approximation (DWBA). Thus, the diffuse scattering factor is then modified to

$$P(\vec{q}) \propto N|F_{DWBA}(\vec{q})|^2 S'(\vec{q})$$

where three additional terms related to reflection before and/or after the scattering event with an object are included. This scattering factor works for the monodisperse ensembles, while the detected objects in the real system are generally not monodisperse. For the simplicity, an assumption based local monodisperse approximation (LMA) is made to model the real system. This assumption describes that scattering factors of different domains are incoherent super positioned together and thus can be treated independently, so these scattering factors are simply summed up as

$$P(\vec{q}) \propto \sum_i N_i \langle |F_i(\vec{q})|^2 \rangle S_i'(\vec{q})$$

As shown in the SEM images, different morphologies, such as cylinders, spheres, and their hybrids, are observed. Therefore, two different shapes are employed for the form factors. The respective form factors are

$$F_s(\vec{q}) = 4\pi R^3 \frac{\sin(qR) - qRcos(qR)}{(qR)^3} exp[iq_z R]$$

for spheres with radius R, and

$$F_c(\vec{q}) = 2\pi H R^2 \frac{J_1(q_r R)}{q_r R} sin\left[(q_z \frac{H}{2}\right] exp\left[iq_z \frac{H}{2}\right]$$

for cylinders with radius R and height H.

The structure factor is related to the mean interdomain distance D and for a one dimensional paracrystalline lattice given by

$$S'(\vec{q}) = \frac{1-\phi^2(\vec{q})}{1+\phi^2(\vec{q})-2\phi(\vec{q})\cos(|\vec{q}|D_c)}$$

where $\phi(\vec{q}) = exp[\pi\omega_D^2 D_c^2 |\vec{q}|^2]$ with ω_D being the standard deviation of a Gaussian distribution at D_c. Since the horizontal cuts are performed at a specific q_z (Yoneda peak), the q_z is constant and approximated as $q_z \approx 0$. The structure factor can then be simplified to

$$S'(\vec{q}) = \frac{1-exp\left[\pi\omega^2 D_c^2 q_y^2\right]^2}{1+exp\left[\pi\omega^2 D_c^2 q_y^2\right]^2 - 2exp\left[\pi\omega^2 D_c^2 q_y^2\right]\cos(q_y D_c)}$$

within the framework of DWBA and LMA, the radii and center-to-center distance of different structures can be resolved by modeling. For data modeling, the low q area is dominated by the resolution function.
